# Novel Single-Nucleotide Polymorphisms (SNPs) and Genetic Studies of the Shadow of Prion Protein (*SPRN*) in Quails

**DOI:** 10.3390/ani14172481

**Published:** 2024-08-26

**Authors:** Da-In Choi, Mohammed Zayed, Byung-Hoon Jeong

**Affiliations:** 1 Korea Zoonosis Research Institute, Jeonbuk National University, 820-120, Hana-ro, Iksan 54531, Republic of Korea; cdi68@jbnu.ac.kr (D.-I.C.); mzayed2@vet.svu.edu.eg (M.Z.); 2 Department of Bioactive Material Sciences, Institute for Molecular Biology and Genetics, Jeonbuk National University, Jeonju 54896, Republic of Korea; 3 Department of Surgery, College of Veterinary Medicine, South Valley University, Qena 83523, Egypt

**Keywords:** quails, prion, *SPRN*, SNP, polymorphism

## Abstract

**Simple Summary:**

The presence of the shadow of prion protein (Sho) produced by the shadow of prion protein gene (*SPRN*) has been correlated with prion disease. In this study, we identified the *SPRN* gene sequence and examined its genetic variations in quails. The quail and chicken Sho amino acid sequences showed 100% identity. A total of 13 novel polymorphisms were found in 106 quails, and three out of four non-synonymous SNPs (A68T, L74P, and M105I) showed deleterious effects on quail Sho. A comparison of genetic polymorphisms in the open reading frame revealed differences in *SPRN* between quails and other mammals and avians. The genetic characteristics of the quail *SPRN* gene are being investigated for the first time in this study.

**Abstract:**

Prion diseases are a group of deadly neurodegenerative disorders caused by the accumulation of the normal prion protein (PrP^C^) into misfolding pathological conformations (PrP^Sc^). The PrP gene is essential for the development of prion diseases. Another candidate implicated in prion pathogenesis is the shadow of the prion protein (*SPRN*) gene. To date, genetic polymorphisms of the *SPRN* gene and the structure of the Sho protein have not been explored in quails. We used polymerase chain reaction (PCR) to amplify the *SPRN* gene sequence and then conducted Sanger DNA sequencing to identify the genetic polymorphisms in quail *SPRN*. Furthermore, we examined the genotype, allele, and haplotype frequencies, and assessed the linkage disequilibrium among the genetic polymorphisms of the *SPRN* gene in quails. Additionally, we used *in silico* programs such as MutPred2, SIFT, MUpro, AMYCO, and SODA to predict the pathogenicity of non-synonymous single-nucleotide polymorphisms (SNPs). Alphafold2 predicted the 3D structure of the Sho protein in quails. The results showed that a total of 13 novel polymorphisms were found in 106 quails, including 4 non-synonymous SNPs. Using SIFT and MUpro *in silico* programs, three out of the four non-synonymous SNPs (A68T, L74P, and M105I) were predicted to have deleterious effects on quail Sho. Furthermore, the 3D structure of quail Sho was predicted to be similar to that of chicken Sho. To our knowledge, this is the first report to investigate the genetic and structural properties of the quail *SPRN* gene.

## 1. Introduction

Prion diseases are characterized as chronic and incurable neurodegenerative disorders in humans and some mammals with no reported treatments [1,2,3]. Prion infection causes a conformational change in the cellular isoform of the prion protein (PrP), resulting in the formation of a pathogenic isoform (PrP^Sc^) that is enriched with beta-sheets and associated with prion infectivity [4]. Although the physiological function of PrP, encoded by the prion protein (*PRNP*) gene, is still not well understood [5], the presence of PrP is important for the incidence of prion disease: *PRNP*-knockout mice have been shown to be resistant to prion infection [6]. However, other genes may influence the pathogenesis of prion diseases.

A member of the *PRNP* family, the shadow of prion protein (*SPRN*) gene, is one of the candidate genes encoding the shadow of prion protein (Sho) [7]. Expression of the *SPRN* gene is specifically limited to the brain, and the Sho protein, a component of the PrP protein family, is expressed in the central nervous system [8]. The Sho protein shares numerous properties with PrP, indicating a possibility of mutual characteristics between the two proteins [9,10]. Studies have suggested that Sho might play a role in modulating the toxicity of prions or in protecting against prion diseases, but further research is needed to clarify its function.

The Sho protein was identified based on its homology to the central hydrophobic region of PrP [10]. Through bioinformatic study of *SPRN* and *PRNP* sequences, it has been suggested that the primordial *PRNP* may be related to *SPRN* and the prion-like protein (*PRND*) gene [11]. Previous studies have reported the consequences of PrP-Sho interactions on the PrP-folding landscape and prion conversion process [12]. Single-nucleotide polymorphisms (SNPs), the most frequent form of genetic variation identified in a species, can seriously affect gene function and phenotypic expression. Because of their prevalence and durability, SNPs are significant markers in genetic research, serving as an important tool for mapping genetic characteristics, analyzing population genetics, and learning about evolutionary processes. With two functional experiments showing the involvement of Sho in prion biology and pathogenesis [8,13], various polymorphisms in *SPRN* have been reported in a genetic test in humans and found to be associated with TSE susceptibility [14]. In addition, a mutation in the entire ovine *SPRN* gene was detected and became known to be associated with susceptibility to classical scrapie type. The vulnerability of prion diseases in various species is associated with genetic variations in *SPRN*, as suggested by these studies.

Studies into prion diseases have been focused on mammals; however, there is an increased interest in investigating the genetics of prions in avian species such as quails. Previous studies on the polymorphisms of the *PRNP* gene have been reported in mammals, birds, and turtles [15,16,17]. Studies on the *SPRN* gene in mammals and birds have also been reported [18,19,20,21,22]. Chickens have shown resistance to prion disease among the several species classified as prion disease-resistant [23]. Recently, *SPRN* polymorphisms have been investigated in two breeds of chickens, Dekalb White and Ross [22]. However, studies on the genetic properties of the *SPRN* gene in quails linked to prion diseases have not been conducted. Quails are widely bred and consumed around the world, similar to chickens. The wild Japanese quail (*Coturnix japonica*) is a well-known terrestrial bird inhabiting grassy fields and indigenous to parts of Asia, such as Japan, China, and Republic of Korea [24]. A previous study of quail genetics found that the microsatellite markers showed significant sequence similarities to those found in chickens [25]. In quails, *PRNP* polymorphisms have been reported in the open reading frame (ORF) region of the quail *PRNP* gene [26]. Exploring the *SPRN* gene in quails in the context of prion diseases could yield valuable insights into the susceptibility of quails to these diseases and potentially shed light on mechanisms of prion transmission across species. To further understand this potential resistance feature in quails, our investigation will focus on genetic and structural characteristics. Here, this is the first report to investigate the genetic and structural properties of the quail *SPRN* gene to our knowledge.

We amplified the sequence of the *SPRN* gene using polymerase chain reaction (PCR) in this study. Additionally, genetic polymorphisms in the *SPRN* gene were discovered through amplicon sequencing. Furthermore, we investigated the frequencies of genotype, allele, and haplotype, as well as the linkage disequilibrium (LD) of SNPs in the quail *SPRN* gene. *In silico* programs were used to predict the impact of non-synonymous SNPs in the quail *SPRN* gene.

## 2. Materials and Methods

### 2.1. Ethical Statement

We collected leg tissue samples from 106 Japanese quails at slaughterhouses in the Republic of Korea. The samples were immediately placed on dry ice and then transported to the laboratory under controlled conditions. Once in the laboratory, the samples were stored at −80 °C until DNA extraction. All experimental procedures related to the quail were approved by the Institutional Animal Care and Use Committee (IACUC) of Jeonbuk National University (JBNU 2021-049).

### 2.2. Genomic DNA

We extracted genomic DNA from 20 mg of peripheral tissue samples from quails using the BioFACT Multi-Bead™ genomic DNA prep kit (BioFACT, Daejeon, Republic of Korea) according to the manufacturer’s guidelines.

### 2.3. Genetic Analysis of the Quail SPRN Gene

**Figure 1:**
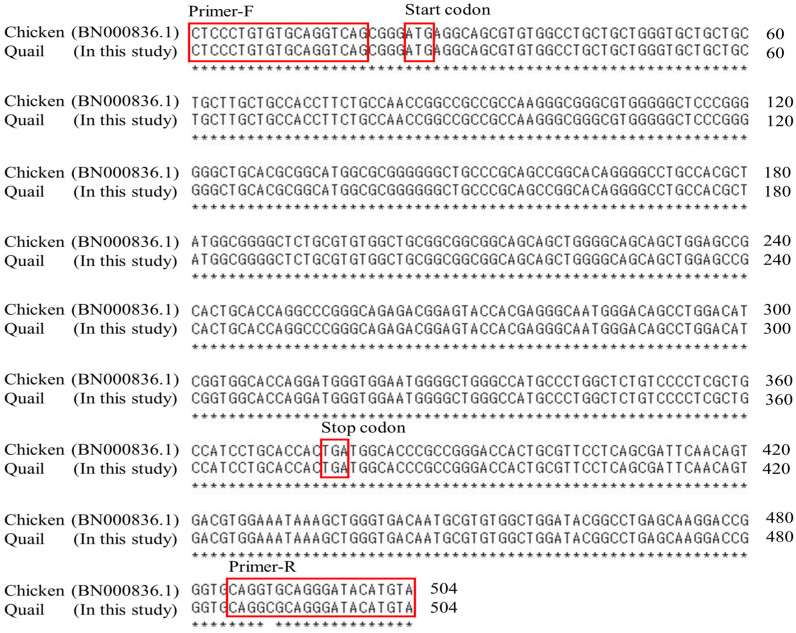
Comparing the gene sequences of the shadow of prion protein (*SPRN*) between chicken (BN000836.1) and quail. The red frame reveals to primer sequences or start and stop codon.

The amplification of the quail *SPRN* gene involved conducting PCR with the *SPRN* forward primer (CTCCCTGTGTGCAGGTCAG) and *SPRN* reverse primer (TACATGTATCCCTGCGCCTG), which were designed based on the chicken *SPRN* gene (Gene ID: BN000836.1). Figure 1 provides detailed information about the primers. The PCR mixture included 2 μL of genomic DNA, 2.5 µL of 10× H-star *Taq* reaction buffer, 2.5 µL of 5× band helper, 1 µL of each 10 mM dNTP mix, 1 µL of each primer (10 µM), and 0.2 µL of H-star *Taq* DNA polymerase (BIOFACT, Daejeon, Republic of Korea). The PCR process involved the following experimental conditions: denaturation at 98 °C for 15 min, followed by 35 cycles of 98 °C for 20 s, 58 °C for 30 s, and 72 °C for 1 min for annealing and extension, and 1 cycle of 72 °C for 5 min for the final extension. PCR was conducted using an S-1000 Thermal Cycler (Bio-Rad, Hercules, CA, USA). In our previous study [26], *PRNP* data were collected to establish the linkage between the *PRNP* and *SPRN* SNPs in quails. To summarize, PCR was utilized to amplify the quail *PRNP* gene using gene-specific primers. These primers, the *PRNP* forward primer (AGGTCTATGCTCGCTGCTCT) and *PRNP* reverse primer (AAGGACAAGGGACACCCCAT), were designed based on the quail *PRNP* gene (Gene ID: 107323677).

### 2.4. Genetic Polymorphisms of the SPRN Gene among Several Species

We collected information on the polymorphisms of the *SPRN* gene from previous studies. Next, we compared and illustrated the polymorphisms using Microsoft PowerPoint version 2407.

### 2.5. The Impact of Amino Acid Substitution by In Silico Evaluation

MutPred2, SIFT, MUpro, AMYCO, and SODA are computational programs that evaluate the impact of non-synonymous SNPs on protein stability or function. MutPred2 software combines genetic and molecular data to make probabilistic decisions about whether amino acid alterations are harmful [27]. SIFT predicts whether an amino acid substitution will change protein function [28]. MUpro is used to predict stability changes of proteins resulting from non-synonymous SNPs [29]. AMYCO is a computational analysis program that shows results as visually appealing ratings ranging from 0 to 1, predicting the likelihood of aggregation into amyloid [30]. Lastly, SODA is a method for predicting variations in protein solubility based on some of the protein’s physico-chemical properties [31].

### 2.6. The 3D Structure Analysis

The 3D structure of Sho in quail was predicted using the AlphaFold2 website (https://colab.research.google.com/github/sokrypton/ColabFold/blob/main/AlphaFold2.ipynb, Accessed on 15 January 2024). Based on a scale ranging from 0 to 100, the predictability of the structure is assessed through the predicted local distance difference test (pLDDT) value. Proposed changes to the structure based on amino acid substitutions were suggested by the Swiss-PdbViewer (https://spdbv.unil.ch/, Accessed on 15 January 2024) [32].

### 2.7. Statistical Analysis

We used Haploview version 4.2 from the Broad Institute in Cambridge, MA, USA, to perform LD and haplotype analysis [33]. The calculation for Hardy–Weinberg equilibrium (HWE) was carried out using the Chi-square test [34].

## 3. Results

### 3.1. SPRN Gene Sequence in Quails

The DNA sequence of the *SPRN* gene in quail has not been previously described. Figure 1 illustrates the designed *SPRN* gene-specific primers by referencing the chicken (*Gallus gallus*) *SPRN* gene sequences. We performed PCR to amplify the ORF region of the quail *SPRN* gene, and the sequencing results were consistent with the registered chicken *SPRN* gene on GenBank (Gene ID: BN000836.1).

### 3.2. Identification of Novel Polymorphisms of the Quail SPRN Gene

**Figure 2:**
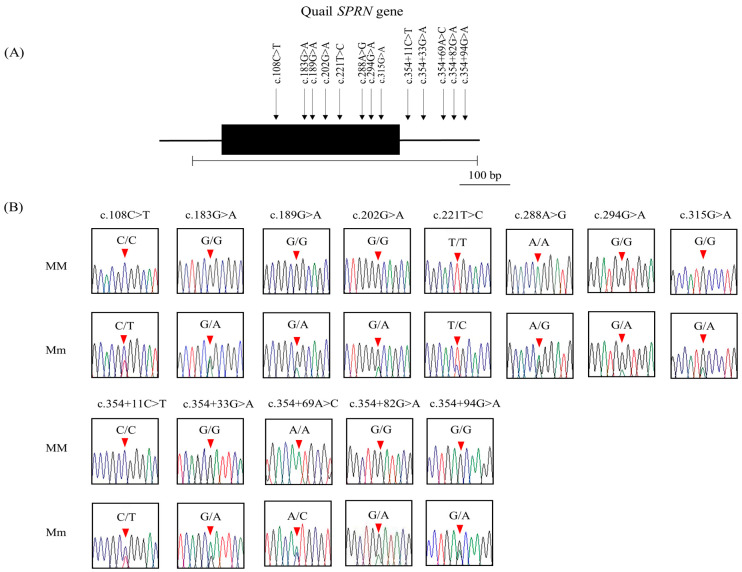
In quails, single-nucleotide polymorphisms (SNPs) were identified in the shadow of prion protein gene (*SPRN*). The *SPRN* gene map and the detected polymorphisms in quails are shown in (**A**). The shaded block marks the open reading frame (ORF) within the exon. Additionally, 13 novel SNPs from this study are indicated by arrows, and the sequenced region is represented by the edged horizontal bar. The electropherograms of the 13 *SPRN* SNPs in quails are depicted in (**B**). The peaks of different colors correspond to each base of the DNA sequence (green: adenine; red: thymine; blue: cytosine; black: guanine). Furthermore, the positions of the SNPs identified in this study are denoted by red arrows. Major homozygotes are indicated as MM, while heterozygotes are denoted as Mm.

In order to detect the genetic variations of the *SPRN* gene in quails, we conducted DNA sequencing on a total of 106 quails. A sum of 13 new SNPs were identified during the process including c.108C>T, c.183G>A, c.189G>A, c.202G>A (A68T), c.221T>C (L74P), c.288A>G, c.294G>A (W98X), and c.315G>A (M105I) in the ORF region; and c.354+11C>T, c.354+33G>A, c.354+69A>C, c.354+82G>A, and c.354+94G>A downstream of the *SPRN* gene (Figure 2 and Table 1). We examined the genotype, allele frequencies, and HWE of the 13 *SPRN* SNPs, as presented in Table 1. With the exception of c.183G>A, c.354+33G>A, and c.354+69A>C, all genetic polymorphisms were found to be in HWE. The LD analysis using r^2^ values (Table 2) revealed seven instances of strong LD (r^2^ > 0.333) among the 13 *SPRN* SNPs. LD between quail *PRNP* and quail *SPRN* SNPs was also studied using r^2^ values, with no strong LD (r^2^ > 0.333) observed between *PRNP* and *SPRN* SNPs in quails (Table 3). Furthermore, haplotype tests were conducted on the 13 SNPs of the quail *SPRN* gene, as shown in Table 4. The most prevalent haplotype was CGGGTAGGCGAGG (50%), followed by CAGGTAGGCACGG (4.2%) and CAGGTGGGCACGG (3.3%).

### 3.3. Comparison of the Number of SNPs in the SPRN Gene among Prion Disease-Related Species

**Figure 3:**
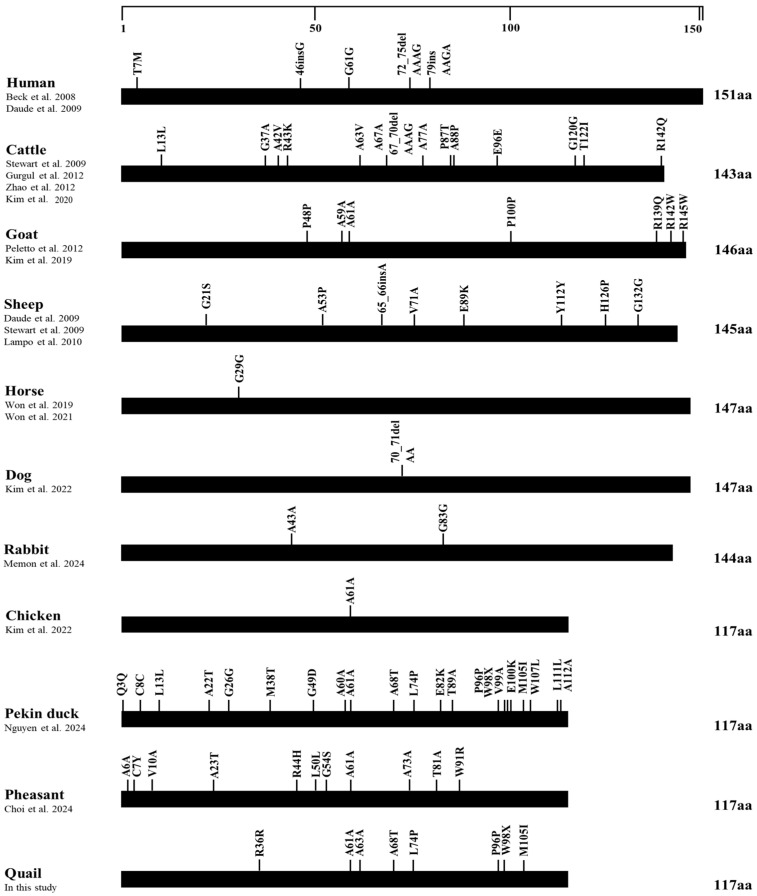
The genetic polymorphisms in the open reading frame (ORF) of the shadow of prion protein gene (*SPRN*) are distributed in various species. The figure shows the documented genetic polymorphisms of the *SPRN* gene in humans [14,35], cattle [36,37,38,39], goats [18,40], sheep [35,36,41], horses [19,20], dogs [21], rabbits [42], chickens [22], Pekin ducks [43], pheasants [44], and quails. The edged horizontal bar means the length of the amino acids in the *SPRN* gene.

In order to identify disparities in SNP numbers between quails and other species, we assembled data on variations in the ORF of the *SPRN* gene from species susceptible to prion diseases (human, cattle, goat, sheep) and those resistant to prion diseases (horse, dog, rabbit, chicken, Pekin duck, pheasant). It is worth noting that the susceptible species exhibited multiple genetic variations resulting in amino acid alterations in the ORF of the *SPRN* gene. Specifically, eight non-synonymous SNPs were discovered in cattle. Goats were found to have three non-synonymous SNPs, while sheep were found to have five non-synonymous SNPs. However, prion disease-resistant species showed fewer non-synonymous changes. Specifically, one synonymous SNP was identified in horses and chickens, and two synonymous SNPs were identified in rabbits. In this study, we identified genetic polymorphisms in quails, including four synonymous and four non-synonymous SNPs (Figure 3).

### 3.4. Polymorphism Distributions among Avian Species

To investigate differences in the ORF in the *SPRN* gene among birds, we analyzed the SNPs of the *SPRN* gene in chickens, Pekin ducks, pheasants, and quails. Previously, we reported that only one synonymous SNP was found in chickens. Interestingly, 21 and 11 polymorphisms were identified within the *SPRN* gene in Pekin ducks and pheasants, respectively. In the current study, we found eight polymorphisms in quails. Pekin ducks were found to have the highest number of polymorphisms, while quails had the second lowest number, following chickens. The several distributions of *SPRN* polymorphisms among avian species are marked in Table 5.

### 3.5. Prediction of the 3D Structure of Quail Sho

**Figure 4:**
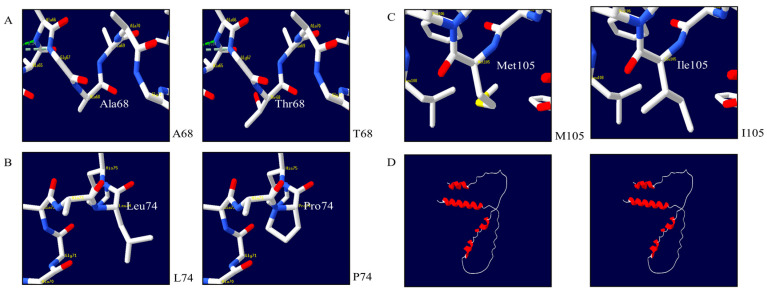
The 3D structure of the shadow of prion protein (Sho) in quails can be predicted. (**A**) shows the 3D structure of quail Sho with the A68 and T68 alleles, as well as the 3D structure of quail Sho with the L74 and P74 alleles (**B**). The 3D structure of quail Sho with the M105 and I105 alleles (**C**). (**D**) The 3D structures of chicken (**left**) and quail (**right**) Sho are compared.

Initially, we created a 3D structural model of the quail Sho protein using AlphaFold2 to investigate the impact of non-synonymous SNPs. Subsequently, we utilized the Swiss-Pdb Viewer software version 4.1.0. to observe alterations in the 3D structure of the quail Sho protein caused by three non-synonymous SNPs (refer to Figure 4). The A68, T68, L74, and P74 alleles did not form hydrogen bonds (Figure 4A,B). Moreover, the M105 and I105 alleles also lacked hydrogen bonds (Figure 4C). Notably, among the observed non-synonymous SNPs is c.294G>A (W98X), which introduces a stop codon. Furthermore, we confirmed that the 3D structures of Sho in chickens and quails are identical. Both animals were predicted to have five alpha helices (Q3-A22, L57-A63, A70, L74-A77, and W107-L115) (Figure 4D).

### 3.6. In Silico Analysis of the Effects of Polymorphisms in the Quail SPRN Gene

To predict the effects on the function and structure of non-synonymous SNPs, MutPred2, SIFT, MUpro, AMYCO, and SODA programs were used (Table 6). Analysis by MutPred2 showed that three non-synonymous SNPs have scores less than 0.5, demonstrating that they are benign. Using SIFT, three non-synonymous SNPs had SIFT scores of 0.0, indicating that they are deleterious. Analysis by MUpro showed that three non-synonymous SNPs had scores less than 0.0, indicating decreased protein stability. AMYCO demonstrated that three non-synonymous SNPs had a score of 0.0, indicating neutrality. We utilized the SODA program to predict protein solubility based on amino acid substitutions. Analyzed by SODA, A68T and M105I variants had scores of −125.746 and −2.04302, respectively, indicating lower solubility. Conversely, the L74P variant had a SODA score of 148.129, indicating higher solubility.

Taken together, the findings demonstrated that a total of 13 novel polymorphisms were identified in 106 quails, including 4 non-synonymous SNPs. Using *in silico* programs, three out of the four non-synonymous SNPs (A68T, L74P, and M105I) were predicted to have a deleterious effect on quail Sho. Additionally, the 3D structure of quail Sho was predicted to be similar to that of chicken Sho.

## 4. Discussion

In this study, we first discovered quail *SPRN* gene sequences. Interestingly, we found 100% identity between the predicted ORF of the *SPRN* gene in chicken and quail. This finding is notable, especially given that a previous study reported only a 96.34% homology between the chicken and quail *PRNP* gene [26]. To analyze susceptibility/resistance factors for prion disease, it is essential to investigate the genetic polymorphism and genes associated with prion disease in various species. Studying polymorphisms may show how genetic variants affect the structure and function of prion-like proteins.

In addition, we discovered 13 unique SNPs, including 4 synonymous and 4 non-synonymous SNPs, within the ORF region of the *SPRN* gene using amplicon sequencing. Among the SNPs identified in quails, no minor homozygotes were found (Table 1). A previous study suggested that embryonic lethality occurs in about 25% of homozygous gene knockouts [45]. Another study reported that knockdown of the *SPRN* gene in PrP-knockout mouse embryos leads to a lethal phenotype [46]. These results suggest that the possibility that the minor homozygotes of *SPRN* SNPs were eliminated due to embryonic lethality cannot be completely ruled out. The four non-synonymous SNPs identified in quails (c.202G>A (A68T), c.221T>C (L74P), c.294G>A (W98X), and c.315G>A (M105I)) are similar to those found in Pekin ducks (Table 5). However, given the diversity of duck breeds, it is essential to extend these comparisons to other breeds such as the mallard. Additionally, due to the variety of bird species, these comparisons should also extend to other bird species. Although the *SPRN* gene is characterized by insertion/deletion (in/del) variations in a G/C-rich tract and missense variation [35], in/del polymorphisms of the *SPRN* gene were not found in quails and other birds (Table 5). Similar results were reported in previous studies, in which we could not observe in/del polymorphisms in prion disease-resistant animals, including horses and rabbits [19,20,42]. However, four in/del polymorphisms were reported in dogs, another prion-resistant animal [21]. *SPRN* gene in/del polymorphisms have been linked to prion disease susceptibility in animals, including cattle, sheep, and goats [18,36,37,38,39,40,41]. Future studies should investigate the association of in/del variants with *SPRN* in prion disease-resistant animals.

To identify the association between *PRNP* and *SPRN* genes, we performed an LD analysis. The results showed that there is no strong LD between *PRNP* and *SPRN* polymorphisms in quails. Horses, being resistant to prion diseases, exhibit limited genetic association between the *PRNP* and *SPRN* genes in comparison to animals susceptible to prion diseases [19]. Therefore, it is needed to ascertain whether these characteristics found in the current study are present in other prion disease-resistant animals.

Using AlphaFold2 and Swiss-PdbViewer, we also assessed the impact of substitutions on the 3D structure and hydrogen bonds of quail Sho. Previous studies have found changes in hydrogen bonds due to variations in birds, such as the Pekin duck and pheasant *SPRN* genes [43,44]. An increasing number of hydrogen bonds can shift the free energy value, potentially affecting protein stability [47]. However, quails did not show changes in the hydrogen bonds with all the non-synonymous SNPs (Figure 4A–C). This characteristic has not been observed in other bird species. Furthermore, to predict the effect of amino acid substitutions, we used several *in silico* programs (Table 6). SIFT predicted that three amino acid substitutions were deleterious, suggesting that those SNPs might affect Sho protein function. MUpro predicted that three amino acid substitutions (A68T, L74P, and M105I) decreased protein stability. It is noteworthy that a single amino acid mutation can considerably alter the stability of a protein structure [29]. The results of SODA analysis demonstrate that A68T and M105I variants had scores of −125.746 and −2.04302, respectively, indicating they are less soluble than wild-type quail Sho. It is important to observe that low protein solubility is a key factor in several human diseases [48,49,50,51]. Therefore, it is critical to underline the genetic mechanisms of the factors that affect protein solubility.

In the field of neuroscience, birds have traditionally served as model organisms for research into neuronal development [52]. Previous studies have reported that the cores of both PrP and Sho contain an evolutionarily conserved, functionally significant domain that aids in neuroprotection [13]. Sho may have a key role in providing PrP-like activity in brain regions lacking PrP^C^. Neuropeptide expression and prediction assessments in quails have been made possible by the sequencing of the chicken genome [53]. Quail is a good model for studying the *SPRN* gene, one of the prion family members. However, our study was limited to focusing on the exon structure of the quail *SPRN* gene. As a bird species, quail appear to exhibit resistance and complete protection against prion diseases, despite reports of prion disease in various mammalian species. More studies are needed to determine the susceptibility of quails to prion diseases and to investigate the possibility that, unlike chickens, they may be a prion disease-susceptible species.

## 5. Conclusions

The aim of the study was to investigate the genetic and structural characteristics of the quail *SPRN* gene to better understand its potential resistance features. In this work, we initially reported quail *SPRN* gene sequences and then detected 13 novel SNPs in the quail *SPRN* gene, including 4 synonymous and 4 non-synonymous variants. *In silico* analysis predicted that three SNPs, excluding c.294G>A (W98X), induce a decrease in protein stability. In addition, the predicted 3D structure of quail Sho is identical to that of the chicken Sho. As far as we know, this is the first analysis of genetic variations in the quail *SPRN* gene.

## Figures and Tables

**Table 1:** The allele frequencies and genotype of the prion protein (*SPRN*) polymorphisms were verified in 106 quails.

Polymorphisms	Genotype Frequencies, *n* (%)			Allele Frequencies, *n* (%)		HWE
	MM	Mm	mm	M	m	
c.108C>T	105 (99.1)	1 (0.9)	0 (0)	211 (99.5)	1 (0.5)	0.9610
c.183G>A	2 (1.9)	104 (98.1)	0 (0)	108 (50.9)	104 (49.1)	<0.0001
c.189G>A	78 (73.6)	28 (26.4)	0 (0)	184 (86.8)	28 (13.2)	0.1171
c.202G>A (A68T)	80 (75.5)	26 (24.5)	0 (0)	186 (87.7)	26 (12.3)	0.1501
c.221T>C (L74P)	78 (73.6)	28 (26.4)	0 (0)	184 (86.8)	28 (13.2)	0.1171
c.288A>G	63 (59.4)	43 (40.6)	0 (0)	169 (79.7)	43 (20.3)	0.0088
c.294G>A (W98X)	78 (73.6)	28 (26.4)	0 (0)	184 (86.8)	28 (13.2)	0.1171
c.315G>A (M105I)	105 (99.1)	1 (0.9)	0 (0)	211 (99.5)	1 (0.5)	0.9610
c.354+11C>T	105 (99.1)	1 (0.9)	0 (0)	211 (99.5)	1 (0.5)	0.9610
c.354+33G>A	2 (1.9)	104 (98.1)	0 (0)	108 (50.9)	104 (49.1)	<0.0001
c.354+69A>C	20 (18.9)	86 (81.1)	0 (0)	126 (59.4)	86 (40.6)	<0.0001
c.354+82G>A	60 (56.6)	46 (43.4)	0 (0)	166 (78.3)	46 (21.7)	0.0043
c.354+94G>A	52 (49.1)	54 (50.9)	0 (0)	158 (74.5)	54 (25.5)	0.0004

X, stop codon. MM, major homozygote; Mm, heterozygote; mm, minor homozygote; M, major allele; m, minor allele. HWE, Hardy–Weinberg equilibrium.

**Table 2:** Analysis of linkage disequilibrium involved the non-synonymous SNPs found in the *PRNP* gene of the quail.

	c.108C>T	c.183G>A	c.189G>A	c.202G>A	c.221T>C	c.288A>G	c.294G>A	c.315G>A	c.354+11C>T	c.354+33G>A	c.354+69A>C	c.354+82G>A	c.354+94G>A
c.108C>T	-												
c.183G>A	0.005	-											
c.189G>A	0.001	0.158	-										
c.202G>A	0.001	0.145	0.058	-									
c.221T>C	0.001	0.158	0.043	**0.415**	-								
c.288A>G	0.001	0.264	0.008	0.036	0.039	-							
c.294G>A	0.001	0.158	0.009	**0.543**	**0.370**	0.039	-						
c.315G>A	0.0	0.005	0.001	0.001	0.001	0.019	0.001	-					
c.354+11C>T	**1.0**	0.005	0.001	0.001	0.001	0.001	0.001	0.0	-				
c.354+33G>A	0.005	**1.0**	0.158	0.145	0.158	0.264	0.158	0.005	0.005	-			
c.354+69A>C	0.007	**0.657**	0.104	0.155	0.082	0.174	0.174	0.007	0.007	**0.657**	-		
c.354+82G>A	0.001	0.288	0.0	0.039	0.042	0.021	0.023	0.017	0.001	0.288	0.189	-	
c.354+94G>A	0.002	0.329	0.051	0.079	0.140	0.087	0.140	0.002	0.002	0.329	0.233	0.014	-

Bold text indicates strong LD (r^2^ > 0.333).

**Table 3:** Analysis of linkage disequilibrium (LD) involving SNPs from *PRNP* and *SPRN* genes, with an r^2^ value, in quails.

	c.108C>T	c.183G>A	c.189G>A	c.202G>A	c.221T>C	c.288A>G	c.294G>A	c.315G>A	c.354+11C>T	c.354+33G>A	c.354+69A>C	c.354+82G>A	c.354+94G>A
c.56C>T (T19I)	0.001	0.003	0.002	0.011	0.011	0.026	0.012	0.001	0.001	0.003	0.0	0.001	0.002
c.61G>A (V21I)	0.001	0.0	0.016	0.001	0.001	0.025	0.002	0.001	0.001	0.0	0.006	0.0	0.004
c.64G>T (A22S)	0.001	0.003	0.001	0.011	0.011	0.017	0.011	0.001	0.001	0.003	0.0	0.003	0.001

Non-synonymous SNPs of the quail *PRNP* gene have been used to analyze LD. The *PRNP* and *SPRN* polymorphisms are represented along the vertical and horizontal axes, respectively.

**Table 4:** The frequency of haplotypes involving 13 *SPRN* polymorphisms in quail was determined.

Haplotype	Frequency, *n* (%)
CGGGTAGGCGAGG	106 (0.500)
CAGGTAGGCACGG	9 (0.042)
CAGGTGGGCACGG	7 (0.033)
CAGGTGGGCACAG	6 (0.028)
CAGACAAGCACGA	6 (0.028)
CAGGTAGGCACAG	5 (0.024)
CAGGTGGGCACGA	5 (0.024)
CAAGTAGGCAAAA	4 (0.019)
CAGGTGGGCACAA	4 (0.019)
CAAGTGGGCACGG	3 (0.014)
CAGGTAGGCAAGA	3 (0.014)
CGGGTAGGCGCGA	2 (0.010)
CAGGTAGGCAAAG	2 (0.010)
Others *	50 (0.235)
Total	212 (1.0)

* Others contain rare haplotypes with a frequency < 0.01.

**Table 5:** The distribution of genetic variations in the open reading frame (ORF) section of the shadow of prion protein (*SPRN*) differs among various avian species.

Species	Polymorphisms	Total	References
Chicken	**Synonymous** c.183G>A **Non-synonymous**	1	Kim et al., 2022 [22].
Pekin duck	**Synonymous** c.9G>A, c.24C>T, c.39G>T, c.78C>T, c.180T>C, c.183G>A, c.288A>G, c.333C>T, c.336T>C. **Non-synonymous** c.64G>A (A22T), c.113T>C (M38T), c.146G>A (G49D), c.202G>A (A68T), c.221T>C (L74P), c.244G>A (E82K), c.265A>G (T89A), c.294G>A (W98X), c.296T>C (V99A), c.298G>A (E100K), c.315G>A (M105I), and c.320G>T (W107L).	21	Nguyen et al., 2024 [43].
Pheasant	**Synonymous** c.18C>T, c.148C>T, c.183A>G, c.219A>G **Non-synonymous** c.20G>A (C7Y), c.29T>C (V10A), c.67G>A (A23T), c.131G>A (R44H), c.160G>A (G54S), c.241A>G (T81A), c.271T>C (W91R).	11	Choi et al., 2024 [44].
Quail	**Synonymous** c.108C>T, c.183G>A, c.189G>A, c.288A>G **Non-synonymous** c.202G>A (A68T), c.221T>C (L74P), c.294G>A (W98X), c.315G>A (M105I)	8	This study

**Table 6:** *In silico* analysis of the impact of non-synonymous single-nucleotide polymorphisms (SNPs) in quail.

Polymorphism	Method	Score	Prediction
c.202G>A (A68T)	MutPred2	0.049	Benign
	SIFT	0.0	Deleterious
	MUpro	−0.672	Decrease
	AMYCO	0.0	Neutral
	SODA	−125.746	Less soluble
c.221T>C (L74P)	MutPred2	0.112	Benign
	SIFT	0.0	Deleterious
	MUpro	−1.964	Decrease
	AMYCO	0.0	Neutral
	SODA	148.129	Soluble
c.315G>A (M105I)	MutPred2	0.030	Benign
	SIFT	0.0	Deleterious
	MUpro	−0.468	Decrease
	AMYCO	0.0	Neutral
	SODA	−2.04302	Less soluble

One of the non-synonymous SNPs, c.294G>A (W98X), is a stop codon.

## Data Availability

All data generated or analyzed during this study are available from the corresponding author upon reasonable request.
